# Imputation of missing daily rainfall data; A comparison between artificial intelligence and statistical techniques

**DOI:** 10.1016/j.mex.2023.102459

**Published:** 2023-10-27

**Authors:** Angkool Wangwongchai, Muhammad Waqas, Porntip Dechpichai, Phyo Thandar Hlaing, Shakeel Ahmad, Usa Wannasingha Humphries

**Affiliations:** a Department of Mathematics, Faculty of Science, King Mongkut's University of Technology Thonburi (KMUTT), Bangkok 10140, Thailand; b The Joint Graduate School of Energy and Environment (JGSEE), King Mongkut's University of Technology Thonburi (KMUTT), Bangkok 10140, Thailand; c Center of Excellence on Energy Technology and Environment (CEE), Ministry of Higher Education, Science, Research and Innovation, Bangkok, Thailand; d Faculty of Environmental Science and Engineering, Kunming University of Science and Technology, Kunming 650500, China; e College of Environmental Science and Engineering, Nankai University, Tianjin 300350, China

**Keywords:** AITs for Imputation missing daily rainfall data, Artificial intelligence, Deep learning, Machine learning, Neural networks, Rainfall, Imputation, Missing data

## Abstract

Handling missing values is a critical component of the data processing in hydrological modeling. The key objective of this research is to assess statistical techniques (STs) and artificial intelligence-based techniques (AITs) for imputing missing daily rainfall values and recommend a methodology applicable to the mountainous terrain of northern Thailand. In this study, 30 years of daily rainfall data was collected from 20 rainfall stations in northern Thailand and randomly 25–35 % of data was deleted from four target stations based on Spearman correlation coefficient between the target and neighboring stations. Imputation models were developed on training and testing datasets and statistically evaluated by mean absolute error (MAE), root mean square error (RMSE), coefficient of determination (R^2^), and correlation coefficient (r). This study used STs, including arithmetic averaging (AA), multiple linear regression (MLR), normal-ratio (NR), nonlinear iterative partial least squares (NIPALS) algorithm, and linear interpolation was used.

• STs results were compared with AITs, including long-short-term-memory recurrent neural network (LSTM-RNN), M5 model tree (M5-MT), multilayer perceptron neural networks (MLPNN), support vector regression with polynomial and radial basis function SVR-poly and SVR-RBF.
• The findings revealed that MLR imputation model achieved an average MAE of 0.98, RMSE of 4.52, and R^2^ was about 79.6 % at all target stations. On the other hand, for the M5-MT model, the average MAE was 0.91, RMSE was about 4.52, and R^2^ was around 79.8 % compared to other STs and AITs. M5-MT was most prominent among AITs. Notably, the MLR technique stood out as a recommended approach due to its ability to deliver good estimation results while offering a transparent mechanism and not necessitating prior knowledge for model creation.

## Graphical abstract

**Figure:**
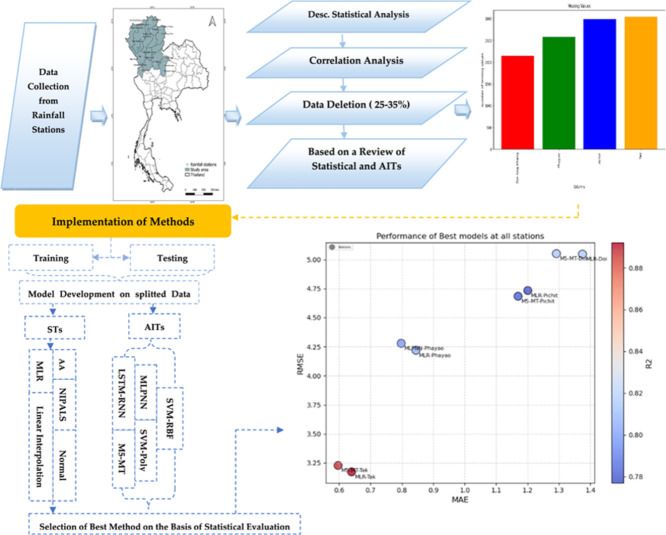

Specifications table

**Table:** 

Subject area:	Engineering
more specific subject area:	modeling and forecasting
Name of your method:	AITs for Imputation missing daily rainfall data
Name and reference of original method:	NA.
Resource availability:	Data used to support the study's findings can be obtained from the corresponding author upon request.

## Introduction

Rainfall is a crucial hydrological factor that initiates various hydrological processes within the system and subsequently provides data for various types of analyses. A comprehensive knowledge of rainfall data is essential for making decisions related to hydrology, global warming and climate change, agriculture, and environmental-related research [1]. The existence of missing data in rainfall datasets poses a pervasive challenge from different sources [2]. Failure to address missing data can lead to compromised analyses, introducing potential inaccuracies and biases [3]. While removing missing time series directly is one method for dealing with missing data, such a procedure may not be viable when the studied weather stations are essential to comprehending specific meteorological processes within the investigated area [1]. Therefore, exploring and employing effective techniques for estimating the missing values becomes imperative, ensuring the attainment of complete and reliable time series data for robust analyses and accurate conclusions. For the imputation of missing rainfall data, numerous authors employed various techniques for imputing the missing values for particular countries or regions based on comparisons to the missing data [4], [5], [6], [7], [8], [9]. So, estimating missing data is optimal and more practical [5]. Many techniques for imputing missing data have been developed. They are classified as statistical to empirical methods and function fitting techniques. Most of these techniques generate the missing values from surrounding station observations. Choosing suitable methods for interpreting missing rainfall data can enhance the precision of hydrological models [10]. In past, multiple statistical techniques (STs) were employed to estimate missing rainfall data, and their selection is contingent upon factors such as the proximity and availability of rainfall data from neighboring stations [8], the duration of data gaps, the extent of available rainfall data, computational demands, and the climatic attributes specific to the study area. Generally, conventional approaches, such as the normal ratio (NR) method, linear interpolation (LI) method, regression-based techniques, and the arithmetic averaging (AA) method, find common utility in the estimation of missing rainfall data, particularly when dealing with relatively limited data gaps [1].

In the past two decades, AITs, including ML (i.e., SVR, random forest), and neural networks (i.e., LSTM-RNN), and decision trees (i.e., M5-MT), have gained significant popularity in hydrological research over the past few decades [11], [12], [13]. These techniques effectively manage the non-linear and uncertain features inherent in hydrological data. Also, showed good results in the imputation of rainfall data. Most statistical analyses and AITs need the use of complete data as compared to data sets that contain missing values. Merely ignoring missing data is an inadvisable approach, as it may result in the loss of valuable data and reduced inferential power [14]. Generally, these imputation methods can be divided into two types based on the dataset used to develop the imputation models. The initial category involves developing an estimation model relying on spatial data. The estimation model is commonly employed to estimate rainfall data missing globally. This is particularly useful in cases where a significant number of rain gauge stations contain missing data and where there is a need to estimate this data concurrently for multiple stations (known as target stations) [15,16]. The second category involves the development of an estimation model that relies on past data from chosen nearest neighboring stations and the target stations. The estimation model is centered on the local scale, employing a limited number of rain gauge stations in the analysis. The current category involves the development of an estimation model that utilizes the historical daily records of rainfall data from chosen nearest neighboring stations and a target station to establish their relationship [4,^17^,18]. This study's research falls under the second category. The following discusses some of the most significant studies using AITs involving estimating and reconstructing missing rainfall data. In Thailand, researchers have addressed the issue of estimating missing daily and monthly rainfall data in various regions by employing machine learning (ML), statistical techniques (STs), and spatial interpolation techniques [12,^19^,20]. Pinthong et al. investigated ML and spatial interpolation methods for estimating missing monthly rainfall data. GP proved effective among ML techniques, while NR performed well among STs. When utilizing these methods, the authors emphasized considering a correlation threshold of 0.80 or higher between the target and neighboring stations and the incidence of missing data is relatively low [12].

While these AITs have contributed to advancing data imputation methods, exploring alternative techniques that can yield superior performance in areas where neighboring rainfall stations are geographically distant and exhibit limited correlation with the target and surrounding observation stations remains necessary.

Such regions pose specific challenges regarding data imputation, prompting further research to identify more effective methodologies for accurate and reliable estimation of missing rainfall data in these circumstances. Therefore, present study also extended previous investigations to encompass the northern regions of Thailand, where the correlation between stations notably diminishes, falling below the threshold of 60 %. Furthermore, the northern region presented challenges in terms of substantial missing daily data, at times reaching levels as high as 35 %. The primary objective of this study is to conduct a comparative analysis of various imputation methods, encompassing AITs such as multilayer perceptron neural network (MLPNN), M5 model tree (M5-MT), long short-term memory recurrent neural network (LSTM-RNN), support vector regression with polynomial kernel (SVR-ploy), and support vector regression with radial basis function kernel (SVR-RBF). Also to compare AITs imputed results with STs, including AA, multiple linear regression analysis (MLR), nonlinear iterative partial least squares (NIPALS) algorithm, and NR and lLI that could effectively address these specific, challenging scenarios in the northern region of Thailand, thus contributing to the field of meteorological data analysis and imputation within such distinct geographical contexts. The comparison is performed on daily rainfall dataset specifically for the Thai Meteorological Department (TMD) target and neighboring rainfall stations. The organizational structure of the manuscript is as follows: the 'Materials and Methods' section includes study area, data acquisition and correlation analysis for the selection of target stations, afterthat imputation techniques STs, and AITs are described. To assess the performance of each method, statistical metrics encompassing coefficient of determination (R^2^), root mean square error (RMSE), mean absolute error (MAE), and the Pearson correlation coefficient (r) are given. Next section “results and discussion” explained the key findings and at the end “conclusion and future directions” provided.

## Materials and methods

### Study area and dataset

**Fig. 1:**
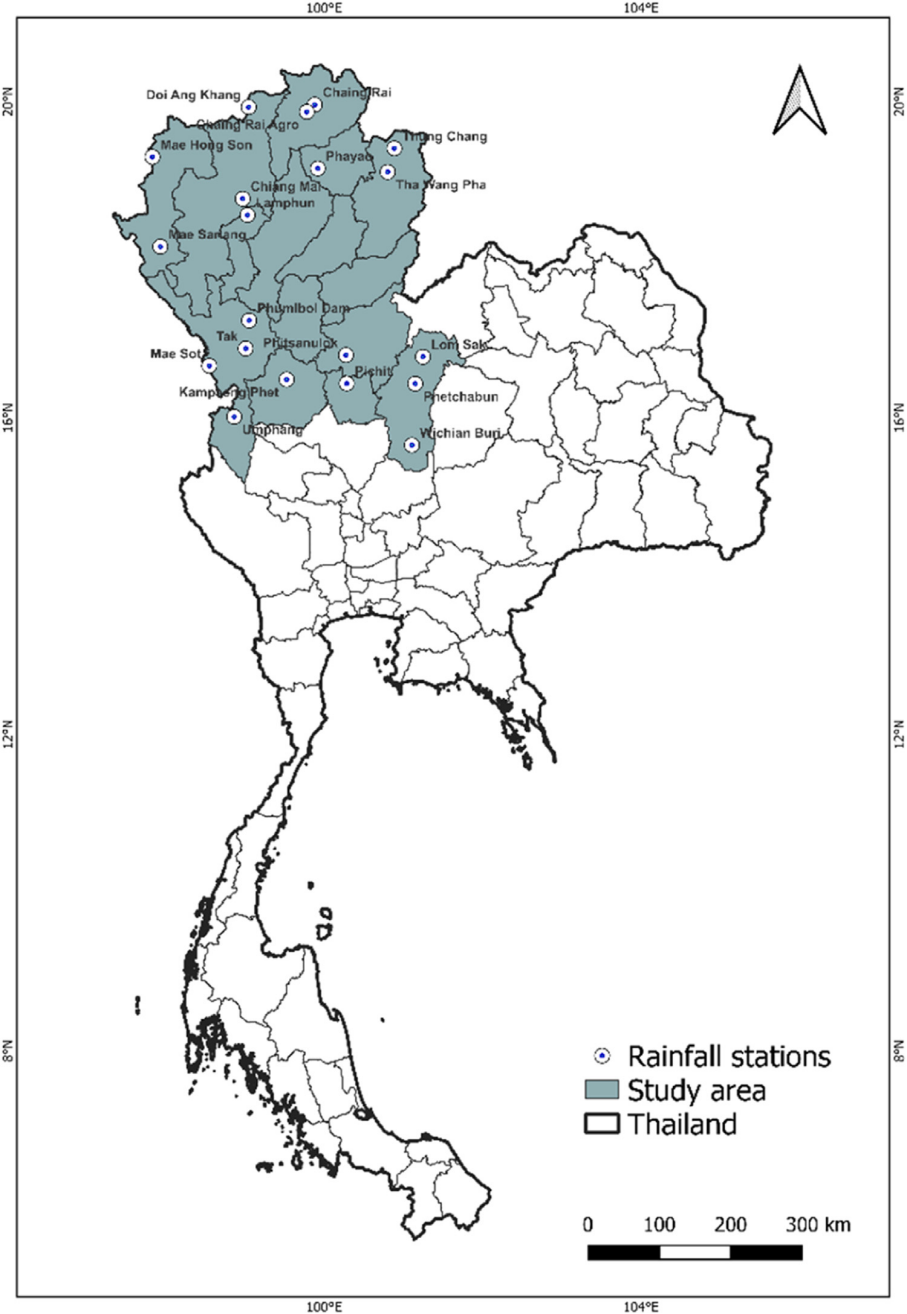
Selected TMD rainfall stations in the northern region of Thailand.

Thailand is situated between 5°37′ and 20°27′ north latitude and 97°22′ and 105°37′ east longitude [21]. Thailand has five climatic regions, each with unique hydrological characteristics that affect the water resources and hydrological processes within each region. This study used 20 TMD rainfall stations from the northern region. The northern region has a tropical monsoon climate with three distinct seasons: a humid season from March to May, a rainy season from June to October, and a cool season from November to February. This region's steep topography and mountainous terrain contribute to rapid drainage and erosion during the wet season [21], [22], [23], [24], [25] Fig. 1.

The present study utilized a dataset comprising 24 years of daily rainfall measurements from 1991 to 2014. Table 1 presents descriptive statistics of daily rainfall at 20 TMD stations. The average rainfall values among the 20 stations vary between 2.84 mm and 4.93 mm. On average, Chaing Rai exhibits the highest mean rainfall, whereas Lamphun experiences the lowest. The data's standard deviation (SD) ranges from 7.96 mm to 12.24 mm. Rainfall stations showing higher SD, such as Chaing Rai and Phayao, demonstrate greater variability in the amounts of rainfall when compared to stations with lower SD, such as Mae Sariang and Wichian Buri. The observed values exhibit a range from 120.6 mm to 259 mm. Umphang shows the most substantial recorded rainfall levels, whereas stations such as Mae Hong Son and Phumibol Dam demonstrate relatively high maximum values.

**Table 1:** Descriptive statistics of all daily rainfall stations in the study area.

**Statistics Parameters**	**Doi Ang Khang**					**Phayao**					**Pichit**					**Tak**				
	**Mae Hong Son**	**Mae Sariang**	**Doi Ang Khang**	**Chiang Mai**	**Lamphun**	**Chaing Rai Agro**	**Chaing Rai**	**Phayao**	**Tha Wang Pha**	**Thung Chang**	**Phitsanulok**	**Phetchabun**	**Pichit**	**Lom Sak**	**Wichian Buri**	**Mae Sot**	**Phumibol Dam**	**Tak**	**Umphang**	**Kampaeng Phet**
**Long**	97.83333	97.93333	99.04833	98.97255	99.03333	99.88139	99.78278	99.92	100.8025	100.8861	100.2759	101.15	100.2855	101.2467	101.1083	98.55083	99.05306	99.00983	98.86556	99.52694
**Lat**	19.3	18.16667	19.93139	18.77127	18.56667	19.96139	19.87083	19.15611	19.11056	19.40806	16.7964	16.43333	16.43777	16.77361	15.65694	16.65917	17.23333	16.87797	16.01583	16.48664
**Mean**	3.71	3.33	4.57	3.18	2.84	4.64	4.93	3.31	4.03	3.75	3.72	3.4	3.35	2.91	3.56	4.28	2.96	3.02	4.22	3.91
**SD**	9.53	7.96	11.72	9.22	8.54	11.67	12.24	9.53	11.24	10.76	10.74	9.31	10.03	8.52	10.35	11.16	9.96	9.64	9.33	10.45
**Min**	0	0	0	0	0	0	0	0	0	0	0	0	0	0	0	0	0	0	0	0
**Max**	128	135	149.4	144.4	156	147.4	147.1	154.3	141.8	259	167.1	143.1	140.8	129.2	125	207.4	247.1	163.5	124.7	120.6

**Fig. 2:**
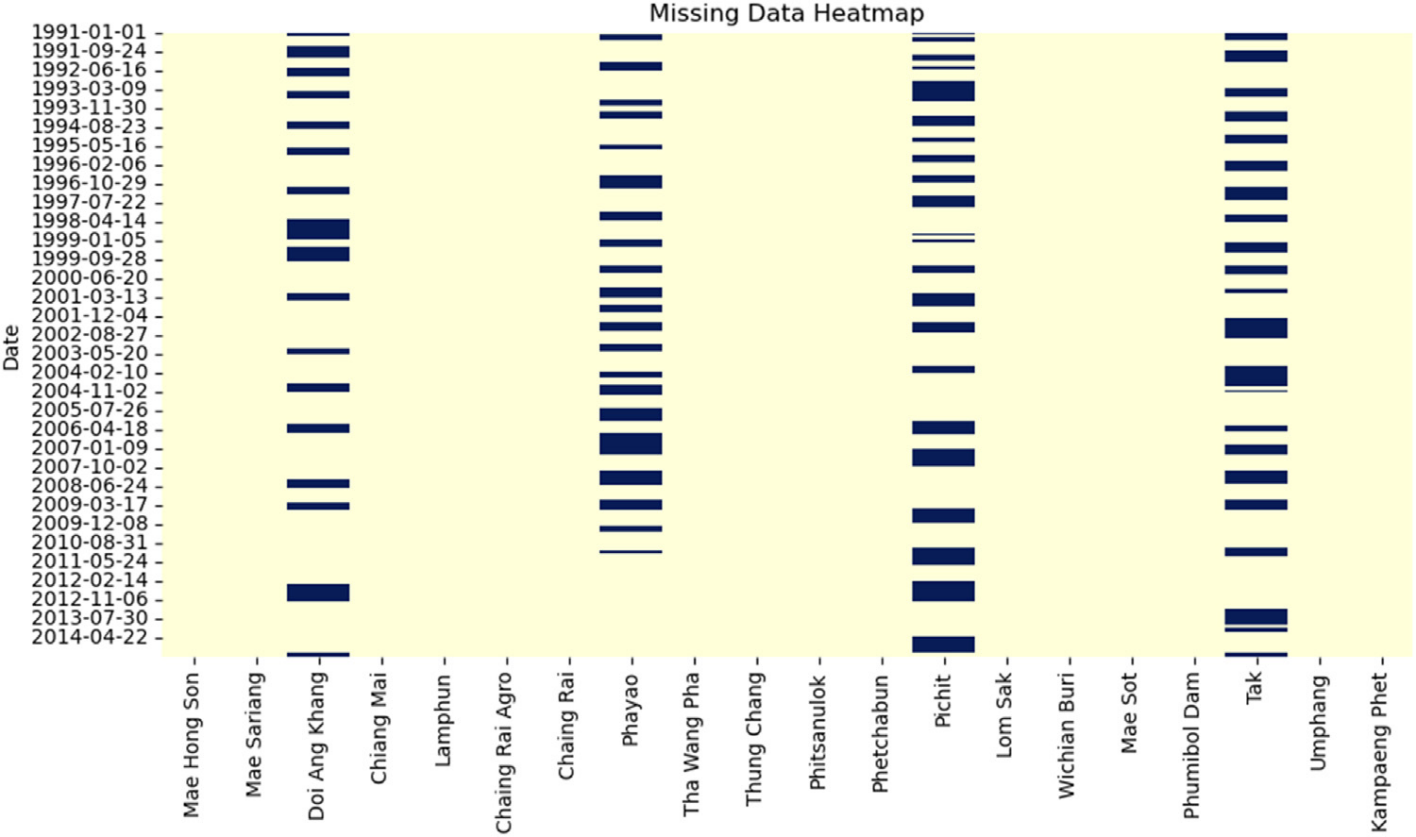
Randomly deleted daily rainfall data from 4 stations (Doi Ang Khang, Phayao, Pichit, and Tak).

**Fig. 3:**
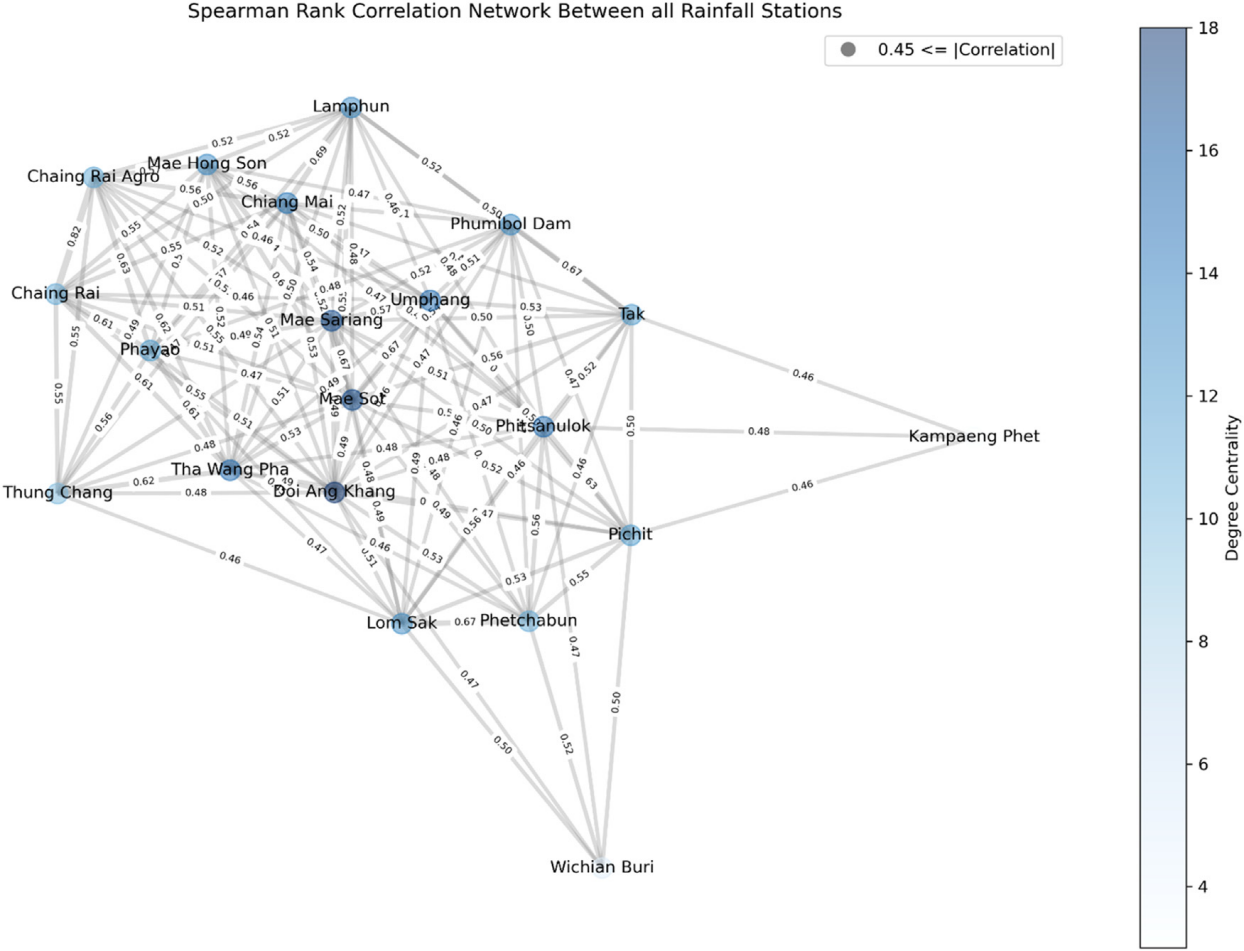
Spearman rank correlation network graph between all rainfall stations.

This study selected four target stations based on the correlation values between target and nieghboring stations to impute missing daily rainfall data, shown in Fig. 3. Fig. 3 represents the Spearman rank correlation analysis and provides important insights into the connections between the study's rainfall stations. The correlation coefficients, which range from −1 to 1, offer insight into the degree of similarity or dissimilarity in the precipitation patterns of variables by providing information on the intensity and direction of monotonic associations between them [26]. The graph's range of degree centrality values, from 4 to 18, represents varied connectedness and influence. Stations with low centrality (4–8) show fewer significant relationships, which could indicate different precipitation patterns. High centrality (13–18) shows stations with significant roles connecting regions, whereas moderate centrality (9–12) indicates localized climatic similarities. Interpreting centrality with correlations and geography reveals information about network dynamics and prominent stations. Positive relationships are prevalent in several geographical areas. For instance, a high correlation between “Mae Hong Son” and “Mae Sariang” of about 0.64 shows regular rainfall patterns. Similarly, “Chiang Mai” and “Lamphun” strongly correlate about 0.69, indicating synchronized rainfall activity. There are moderate relationships between specific stations, showing that they share local climate influences. “Doi Ang Khang” and “Chiang Rai Agro” are two stations that have moderate correlations with many nearby stations, probably due to comparable environmental circumstances. On the other hand, some stations show weak associations, indicating autonomous precipitation behavior. For example, the correlation between “Phayao” and “Phetchabun” is roughly 0.44, indicating a wide range of hydrological patterns. Based on this correlation coefficient results, target rainfall stations (Doi Ang Khang, Phayao, Pichit and Tak) were selected. Fig. 2 shows the missing portion indicates the proportion of missing data for all stations. Among the four target stations where data was randomly removed, Tak has the highest percentage of missing portions, 34.74 %. Phayao and Pichit have a missing percentage of 29.43 % and 34.09 %, respectively, followed by Doi Ang Khang at 24.47 %.

**Fig. 4:**
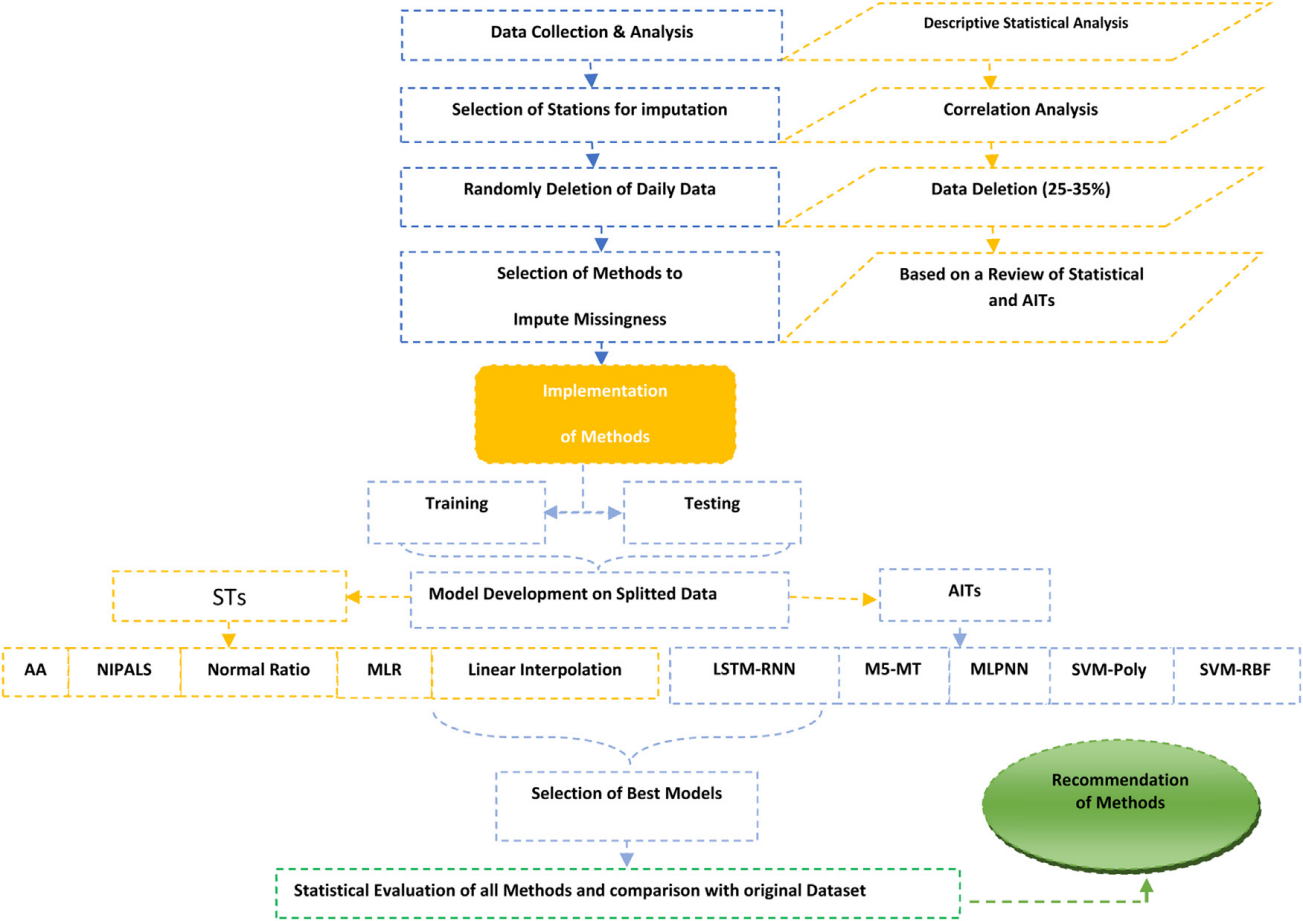
Overall methodology for imputation of missing daily rainfall data.

This study used a comprehensive methodology that combined STs and AITs to impute missing daily rainfall data. The first step is to gather data on observed rainfall, which is then statistically examined to determine its characteristics and patterns of distribution. All stations are subjected to correlation analysis to determine which ones are best for imputing missing data based on their similarities. About 25–35 % of the daily data is randomly deleted to simulate missingness. Various STs are included in imputation methods, such as AA, NIPALS, MLR, LI, and the NR method. AITs, including LSTM-RNN, M5-MT, MLPNN, SVM-Poly, and SVM-RBF, are compared with the STs. The missing rainfall data are inputted using methods and the available observed values. Statistical metrics (R^2^, RMSE, MAE, and r) are used to evaluate the imputation results from each method. These accuracy, precision, and error metrics are computed to assess how well the imputation techniques perform. These evaluations help identify the best imputation technique and reveal the most efficient way to handle missing daily rainfall data. A detailed description of these methods is given below. The overall methodology for handling missing data is shown in Fig. 4.

## Statistical techniques (STs)

### Arithmetic averaging (AA)

This simple technique is often used in meteorology to fill in missing weather data. Calculating the mean of the dataset correlating to the nearest rainfall stations yields the missing data, as illustrated in Eq. (1).

(1) $$R_{o}=\frac{\sum_{i=1}^{n}R_{i}}{N}$$

R_o_ is the missing data at the target station, R_i_ is the daily rainfall at the nearest stations, and N is the total number of rainfall stations closest to the point of interest. The AA technique is acceptable if the rainfall stations are scattered consistently across the area, and particular station values do not deviate significantly from the mean [26].

Multiple linear regression (MLR)

The MLR is a statistical technique used to determine the optimal combination of independent variables that can effectively predict the dependent variable, also known as the criterion variable [27]. Eischeid et al. (1995) elucidated numerous benefits of this approach in estimating missing data [28]. Estimating the missing rainfall data at the target station (Ro) is derived from the formula:

(2) $$R_{o}=\;a_{o}\;+\;\sum_{i=1}^{n}\left(a_{i}*R_{i}\right)$$

where a_i_, *i* = 1, 2, 3, 4, …. n are the regression coefficients, and R_i_ is the daily rainfall at the nearest stations.

### Nonlinear iterative partial least squares (NIPALS) algorithm

Ref. [29] first presented the NIPALS algorithm under NILES. It uses principal component analysis iteratively to the dataset with missing values. The primary objective is to compute the slope of the least-squares line that passes the origin of the observed data points. The variation of the NIPALS components is used to reveal the eigenvalues. The missing data can be anticipated using this method. The pace of the algorithm's convergence is proportional to the proportion of missing data [26].

### Normal-Ratio (NR) method

The normal ratio (NR) is a recommended method for estimating missing data. This method calculates the mean ratio between a station with missing data and other stations where the corresponding data is available [30]. The following formula determines the calculation of the missing value:

(3) $$R_{o}=\frac{\sum_{i=1}^{n}\frac{\mu x}{\mu i}\;*\;R_{i}}{n}$$

R_o_ represents the estimated value of the missing data for the target station. R_i_ denotes the rainfall data of the ith nearest station. µx and µi represent the mean annual rainfall values of station x and the ith nearest station, respectively and “n” represents the number of adjacent stations.

### Linear Interpolation (LI)

Linear interpolation (LI) is used to estimate daily rainfall data at target stations that demonstrate similar conditions. The process entails establishing a linear connection between the target and neighboring data points, which is then utilized to estimate the absent value by considering its relative position along the axis. The methodology assumes a linear correlation between the established data points and offers a straightforward yet reasonably precise estimation for the undisclosed value [31]. The formula for LI can be expressed as:

(4) $$y=\;y_{1}+\left(x-x_{1}\right)\frac{\left(y_{2}-y_{1}\right)}{\left(x_{2}-x_{1}\right)}$$

“y” represents the interpolated value at target station, “x” is the position along the x-axis where missing value is interpolated, x_1_ and x_2_ are x-values before and after the position of “x.” “y_1_” and “y_2_” are the corresponding values of rainfall values before and after the position of “x.”

## Artificial intelligence-based techniques (AITs)

### Multilayer perceptron neural network (MLPNN)

The MLPNN model can be conceptualized as a semi-parametric nonlinear function that establishes a relationship between the input and output data. This approach has been extensively employed to represent intricate associations among datasets [32]. The MLPNN approach uses neighboring stations to estimate missing values [33]. It uses multilayers of neurons to acquire an in-depth knowledge of intricate associations among established data points. By leveraging input from neighboring stations, it generates predictions for the absent value [12,^34^,35]. The MLP architecture consists of an input layer, hidden layer(s), and an output layer with interconnected neurons. The synaptic weights between neurons are manipulated during the learning process using trial and error. The number of hidden layers and neurons is determined based on minimizing deviations between the output and actual values through iterative weight adjustments using a learning algorithm. The neuron's activation function can be linear or non-linear, and the learning process aims to minimize the differences between predicted and observed values [32,36]. The mathematical representation of a MLPNN for estimation is expressed as follows:

(5) $$\hat{R}{\;}_{\text{target}\;\text{station}}\;=F_{\text{output}}\;\left[\sum_{j=1}^{m}W_{j}F_{\text{hidden}\;\text{layer}}\;\left(\sum_{i=1}^{n}w_{\text{ii}}*\;R_{i}\;+\;\alpha_{o}\right)+\beta_{o}\right]$$

The weights denoted by wji represent the connections between the inputs and the hidden layer, while the weights denoted by wj represent the connections between the hidden layer and the output layer. The threshold values, αo and βo, are biases in the system.

### Support vector regression (SVR)

The Support Vector Machine (SVM) is a versatile algorithm for classification and estimation tasks. For classification, SVM minimizes classification errors, while for regression, it aims to minimize fitting errors in data [37]. SVR is a regression technique directly derived from the theory of SVM, as proposed by [38].

In this study, we adopted the method employed by [12]. The SVR aims to predict a linear correlation between the input vector (x ∈ Rn) and the output variable (y ∈ R) in n-dimensional real number space. During training, SVR determines optimal weights and biases using input and output data from the training dataset. Subsequently, these obtained weights and biases are employed to estimate output results for new input datasets based on the established training. SVR fulfills two primary functions: evaluating prediction errors during training and computing output values by considering weights, biases, and input data [12].

(6) $$f\left(x\right)\;=\;\sum_{i=1}^{j}\left(\alpha_{i}-\alpha_{i}^{*}\right)*k\left(x_{i},x_{j}\right)\;+\;b$$

Where b is the bias, $\alpha i,\;\alpha i^{*}$ are Lagrange multipliers, and k (xi,xj) is the Kernel function. The popular Kernel functions used in this study are mathematically presented as follows:

(i) SVR-Poly
  (7) $$k\left(x_{i},x_{j}\right)=\;{\left(1+\;x_{i}\;*\;x_{j}\right)}^{d}$$
(ii) SVR-RBF
  (8) $$k\;\left(x_{i},x_{j}\right)=\text{exp}\left(-\frac{{∥x_{i}-x_{j}∥}^{2}}{2\alpha^{2}}\right)$$

### M5 model tree (M5-MT)

The M5-MT is a variation of the model created by [39] in which linear functions are used at the leaves instead of discrete class labels [40]. The M5 model employs a divide-and-overcome strategy, proceeding from the top toward the bottom of the tree [41]. This dividing criterion is determined by the standard deviation reduction (SDR) formula (8):

(9) $$\text{SDR}=\;SD_{\left(t\right)}-\sum_{i=0}^{n}\frac{\left|t_{i}\right|}{\left|t\right|}\;*SD_{\left(\text{ti}\right)}$$

"t" is the group of samples that reach the node, ti is the subset of samples with the ith possible outcome, and sd is the standard deviation. The application of this procedure reduces the standard deviation of child nodes. The model selects the ultimate split to maximize expected error reduction [39]. The model may become excessively substantial due to test data overfitting.

### Long short-term memory recurrent neural network (LSTM-RNN)

Recurrent neural networks (RNNs), such as LSTM and gated recurrent units [42], have been demonstrated to attain state-of-the-art performance in various real-world applications with multivariate time series data by constructing deep hierarchical features. Furthermore, they can capture important long-range correlations in time series data. Recent attempts to address missingness in RNNs have included concatenating missing entries, incorporating a time-based decay function, and corresponding distinct sampling frequencies. Missing values are a significant problem frequently occurring in time series data (e.g., Meteorological observation data) [43]. LSTM is a modified variant of RNN that overcomes the vanishing gradients problem by permitting forgetting or retaining information for each state [44]. LSTM-RNNs are well-suited for capturing long-term dependencies and patterns in sequential data, effectively predicting missing values in daily rainfall records. By analyzing historical rainfall data from neighboring stations, the LSTM-RNN can learn temporal patterns and relationships to estimate missing values accurately. The approach offers a robust and efficient solution for filling gaps in daily rainfall datasets, enabling better hydrological analysis and forecasting [45].

### Evaluation metrics

This study employs the R^2^, RMSE, MAE, and correlation (r) statistical evaluation metrics to evaluate the model's suitability. These four statistical parameters' formulae are as follows:

(10) $$R2=1-\sum \frac{\left(R_{\text{obs}}\;-\;R_{\text{pre}}\right)\wedge 2}{\left(R_{\text{obs}}-R_{\text{avg}}\right)\wedge 2}$$

(11) $$\text{RMSE}=\sqrt{\sum \frac{{\left(R_{\text{obs}}-\;R_{\text{pre}}\right)}^{2}}{N}}$$

(12) $$\text{MAE}=\sum \frac{\left(R_{\text{pre}}\;-\;R_{\text{obs}}\right)}{N}$$

(13) $$r=\frac{n\left(\sum \text{xy}\right)\;-\;\left(\sum x\right)\left(\sum y\right)}{\sqrt{\left[n\sum x^{2}\;-\;\left(\sum y^{2}\right)\right][n\sum y^{2}-\left(\sum y^{2}\right)}}$$

The R^2^ value ranges from 0 to 1 for an effective relationship between predicted and observed values. The model is considered the most effective when the R^2^ value is close to or equal to one. The RMSE ranges from 0 to for model efficiency. A lower RMSE number suggests a good model, whereas a greater one indicates a poor model or dataset [46], [47], [48]. A correlation (r) might be anything between −1.00 and +1.00. A score of −1.00 represents a perfect negative correlation, whereas a score of +1.00 represents a perfect positive correlation, and 0.00 shows no relationship exists between the variables under examination [49].

## Results and discussion

**Fig. 5:**
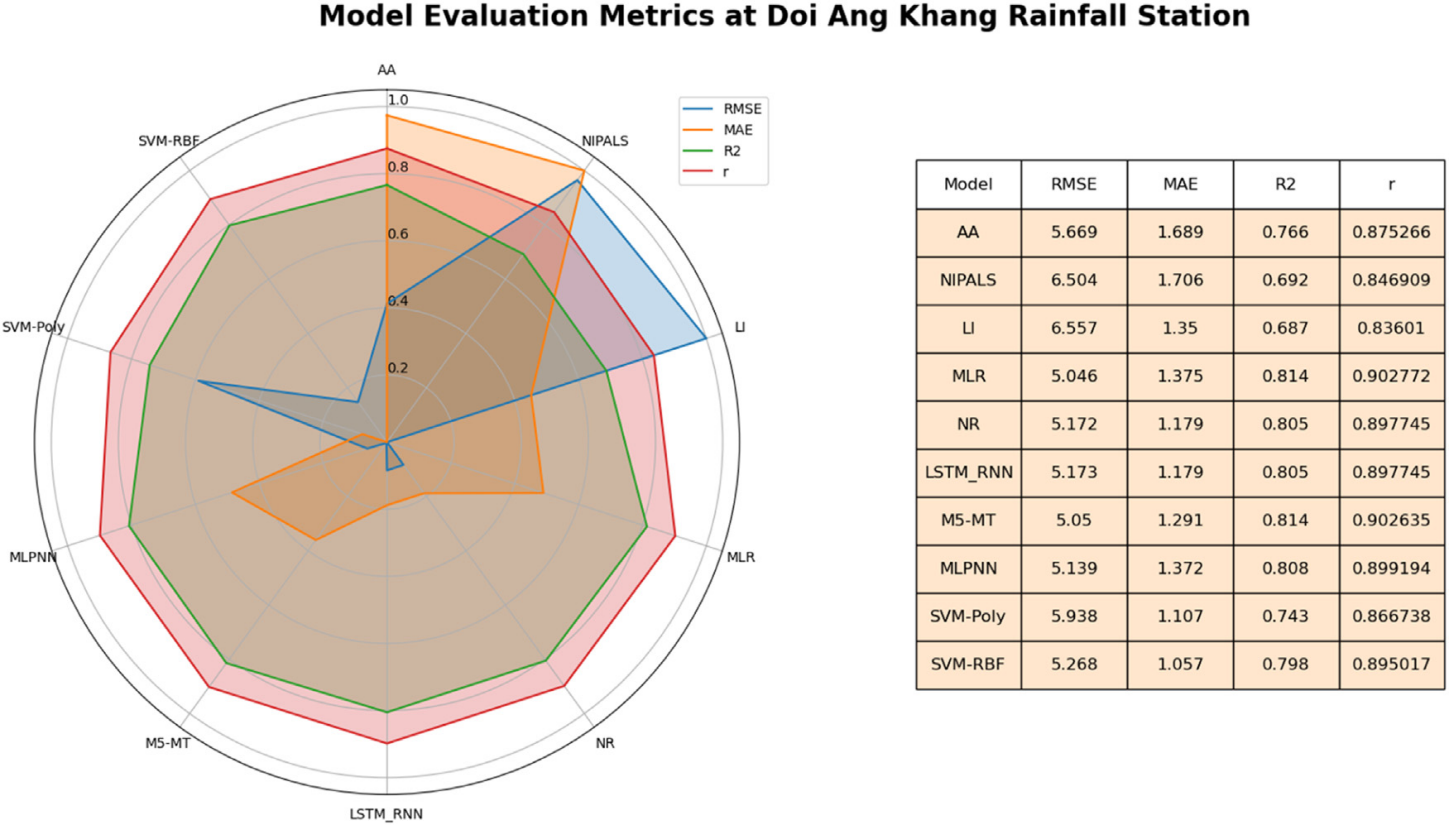
Performance of imputation methods at Doi Ang Khang rainfall station.

This study evaluated various imputation models for imputing missing daily rainfall data. The models were assessed based on four key metrics: MAE, RMSE, R^2,^ and correlation (r). For the graphical representation of results this study used radar mapswhich is graphical tool that displays multivariate data in a two-dimensional chart with multiple axes emanating from a central point [50,51]. Each axis on the map reflects a statistic (RMSE, MAE, R^2^, Correlation). A data point on the chart represents imputation methods. The value of the related metric for that approach is indicated by the data point's distance from the center along each axis. The curve generated by connecting the data points of each method represents how well that method performed across the various measures. This representation is used to identify patterns, trends, and outliers that might not be immediately apparent when examining numerical values alone [51]. Results At Doi Ang Khang rainfall station, MLR and the NR method performed well among the STs, as shown in Fig. 5. The MLR and NR models exhibited strong performance across various metrics, including a relatively low RMSE of 5.046 and 5.172 respectively signifying their ability to minimize imputation errors. Additionally, both models achieved a high R^2^ value of 0.81 as compared to other STs. This high R^2^ value suggests that the MLR model's imputed values align well with the observed data distribution. The model's correlation coefficient (r) between actual and imputed values was 0.90 which underscores their effectiveness in approximating the relationship between neighboring rainfall stations. Similarly, the M5-MT model demonstrates favorable outcomes with a competitive RMSE (5.05), MAE (1.29), r (0.90) and an R^2^ value of 0.81 as compared to other AITs. These metrics emphasize the M5-MT model's proficiency in generating imputations that closely align with rainfall observations. Furthermore, the LSTM-RNN models showcase comparable results, exhibiting a shared RMSE of 5.173 and an R^2^ value of 0.81.

This performance congruence underscores that among STs NR and MLR models' reliability in estimating missing rainfall data. Comparatively, the LSTM-RNN and M5-MT models outperformed the other AITs i.e., MLPNN, SVM-RBF and SVM-Poly in terms of RMSE and R^2^. For Doi Ang Khang, the NR, MLR among STs and M5-MT and LSTM-RNN are most reliable techniques for imputing missing daily rainfall dataset.

The radar map in Fig. 5 graphically illustrates the performance of various methods in imputing missing daily rainfall data across multiple metrics. Upon careful analysis, several noteworthy patterns emerge. Firstly, among STs the MLR and NR takes center stage, showcasing a remarkably well-rounded performance profile. Their data points extend outward on all axes, signifying an impressive balance between minimizing MAE, r and RMSE while achieving a high R^2^. This underscores MLR and NR's proficiency in accurately predicting rainfall and capturing the underlying variability in the data. Further enhancing the Radar Map, M5-MT and LSTM-RNN exhibits a shape akin to MLR, with data points stretching outward across all axes. This robust pattern indicates M5-MT and LSTM-RNN's commendable performance, promising precise predictions, a robust fit (high R^2^), and diminished errors (low MAE and RMSE).

Beyond these prominent methods, the remaining approaches manifest diverse shapes on the Radar Map, pointing toward specific strengths across certain metrics while potentially compromising performance in others. Noteworthy among these is SVM-Poly, displaying an outward extension on the MAE axis, emblematic of its accurate predictions. Conversely, SVM-RBF exhibits prowess in capturing variability, as highlighted by its extension on the R^2^ axis, yet it may contend with comparatively higher errors (MAE, RMSE).

Based on a comprehensive assessment encompassing statistical metrics, the MLR, NR (STs) and M5-MT and LSTM-RNN (AITs)emerged as promising methods for imputing missing daily rainfall data at the Doi Ang Khang station. Their consistent alignment with observed data positions these models as robust solutions for addressing missing data challenges in hydrological contexts.

**Fig. 6:**
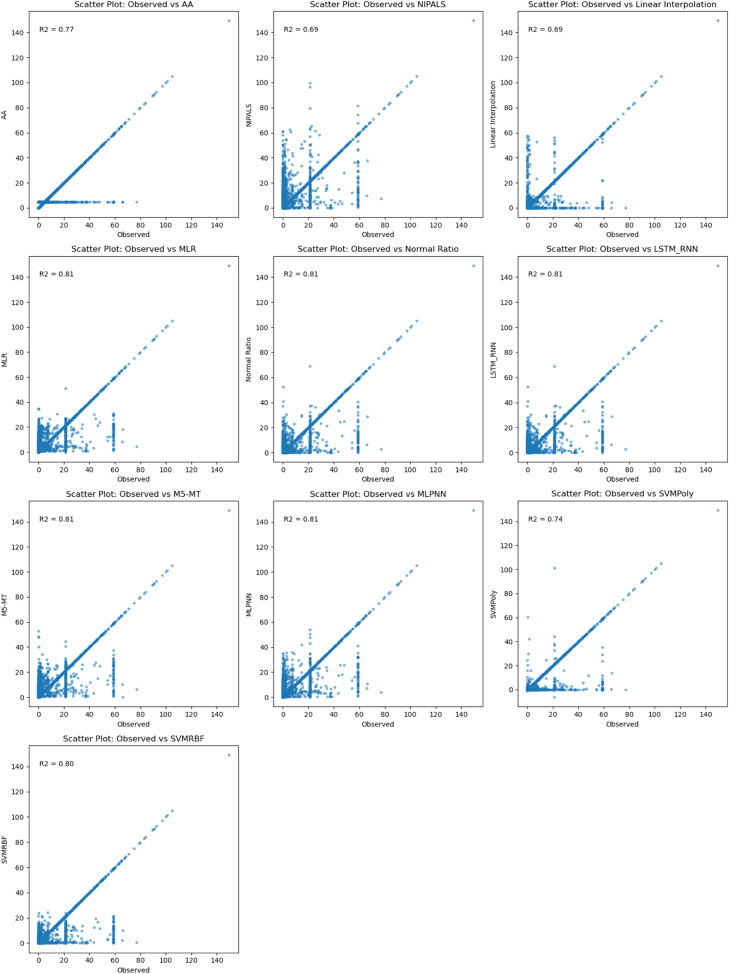
Comparison between observed and results of imputation methods at Doi Ang Khang rainfall station.

In Fig. 6, among the evaluated imputation models for estimating missing rainfall data at Doi Ang Khang rainfall station, two top-performing models based on R^2^MLR, NR, M5-MT, and LSTM-RNNdemonstrated excellent performance, achieving the approximately same high R^2^ value of 0.81. These results indicate a strong correlation between the observed and predicted rainfall data at the Doi Ang Khang rainfall station. The models effectively capture the underlying rainfall patterns and provide accurate imputations of missing values. These models offer reliable and accurate imputations, improving the quality and reliability of hydrological analyses and related studies in the specific Doi Ang Khang rainfall station context.

**Fig. 7:**
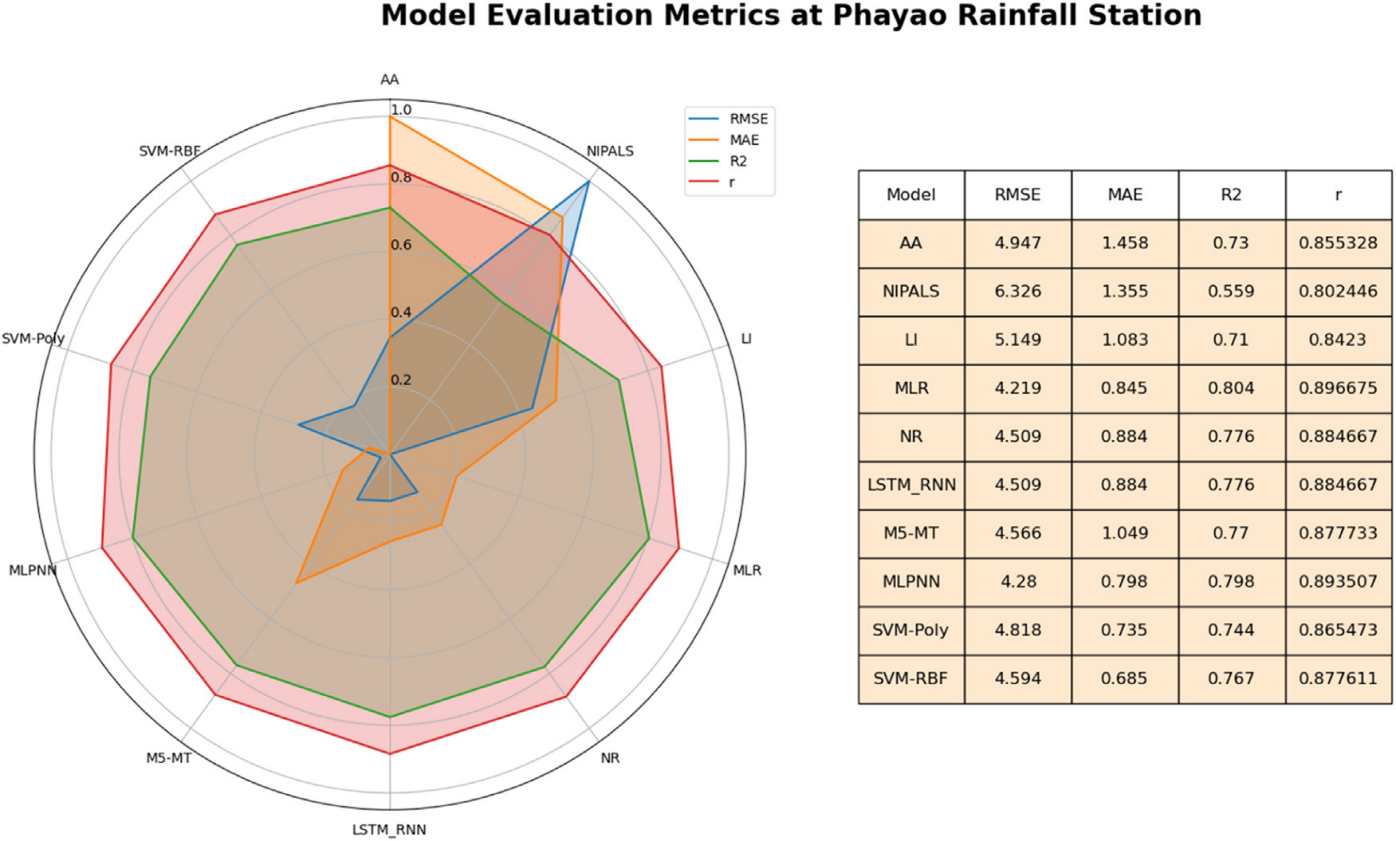
Performance of imputation methods at Phayao rainfall station.

At Phayao rainfall station, it can be seen in Fig. 7 that MLR exhibited exceptional accuracy among the STs. The MLR model emerges as a strong contender, exhibiting remarkable outcomes across various metrics. With a notably low RMSE of 4.218 and a high R^2^ value of 0.80, the MLR model showcases its proficiency in minimizing imputation errors while effectively capturing the variance within the observed data. Moreover, the model's correlation coefficient (r) of 0.896 signifies its capability to establish meaningful associations. Similarly, the M5-MT model demonstrates notable performance with a competitive RMSE of 4.566 and an R^2^ value of 0.77.

Furthermore, the MLPNN model merits attention, boasting a low RMSE of 4.279 and a noteworthy R^2^ value of 0.79.It is worth noting that the NR and LSTM-RNN models exhibit consistent outcomes, both delivering an RMSE of 4.508 and an R^2^ value of 0.776. The efficacy of other STs (AA, LI, NIPALS) and AITs (SVM-RBF, SVM-Poly, and M5-MT) was observed to exhibit relatively diminished levels of accuracy in comparison to the MLR and MLPNN methods. Overall, at Phayao rainfall station, MLR and MLPNN both model's prediction was accurate in imputation missing values.

MLR and MLPNN stand out in Fig. 7 at Phayao station. MLR data points stretch outward across all axes, demonstrating its ability to reduce MAE and RMSE while maintaining a high R^2^. M5-MT exhibits outward expansions on the MAE and RMSE axes, indicating their ability to minimize mistakes and generate solid predictions. These extensions, however, are substantially shorter on the R^2^ axis, implying that while they capture variability, their predictive strength may fall short of MLR. The MLPNN approach is like MLR, with data points stretching outward across all axes. It highlights MLPNN's outstanding performance in making accurate predictions, providing a strong fit (high R^2^), and delivering decreased errors (low MAE and RMSE). While every method has various strengths, a few have specific characteristics. SVM-Poly outperforms in terms of minimizing MAE, demonstrating its ability to provide highly accurate predictions. Conversely, SVM-RBF effectively captures variability, as evidenced by its expansion on the R^2^ axis.

In summary, The MLR and MLPNN models are particularly effective strategies for imputing missing daily rainfall data at the Phayao station. While other methods excel in some areas, these three provide accurate forecasts, a good fit for the data, and relatively low errors.

**Fig. 8:**
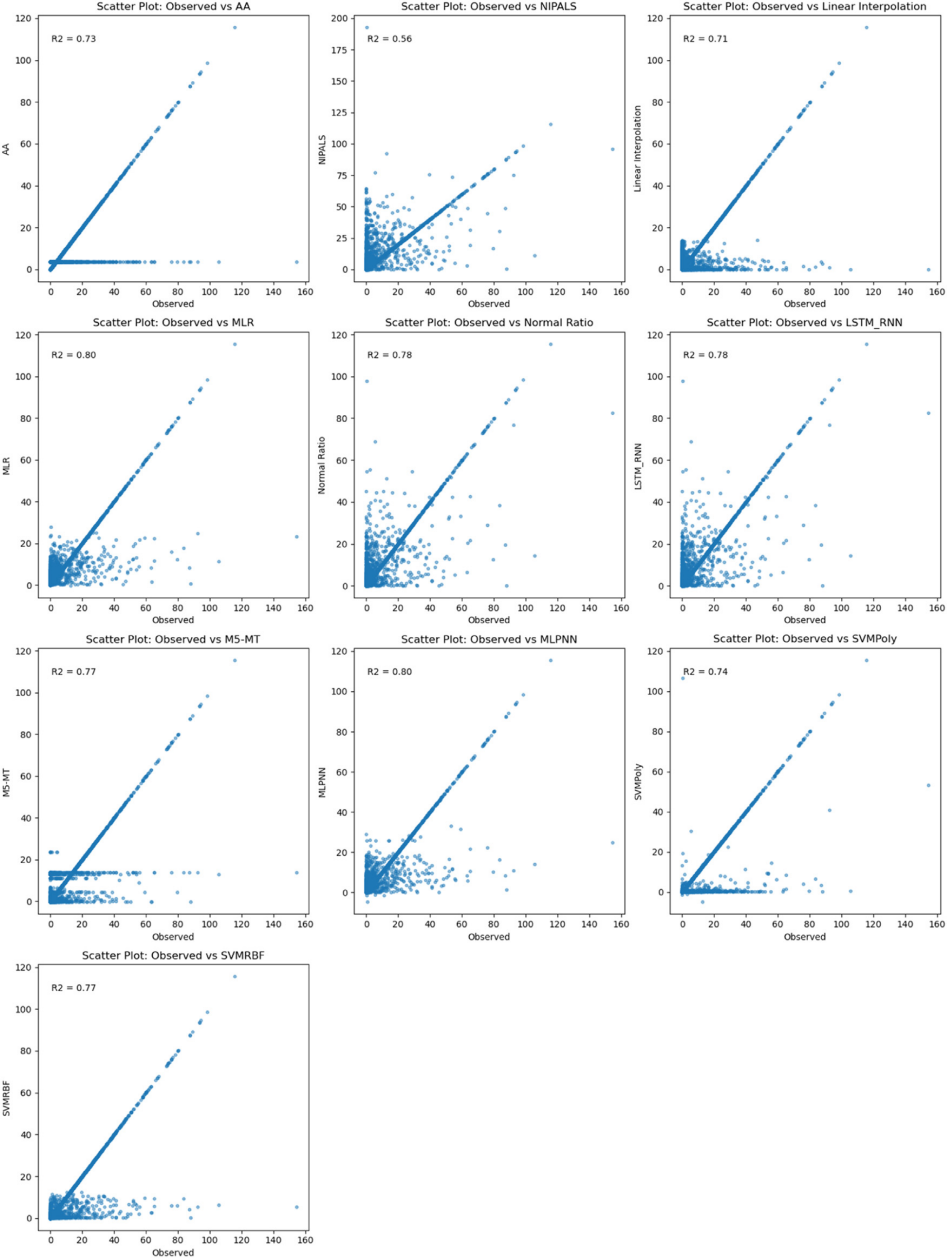
Comparison between observed and results of the imputation method at Phayao rainfall station.

In Fig. 8, The R^2^ values between the observed and predicted missing rainfall data at Phayao Rainfall station, utilizing data from nearby stations, were evaluated for various imputation methods. Among the methods tested, MLR and MLPNN achieved the highest R^2^ values of 0.80, indicating a strong correlation between the observed and predicted data. The Normal method, LSTM-RNN, and M5-MT also exhibited favorable R^2^ values of 0.78 and 0.77, suggesting a good fit of the imputation models. NIPALS displayed the lowest R^2^ value of 0.56, indicating a weaker relationship between the observed and predicted data. These findings emphasize the effectiveness of MLR, MLPNN, and other methods in accurately estimating missing rainfall values at the Phayao Rainfall station, assisting in hydrological analyses and related studies.

**Fig. 9:**
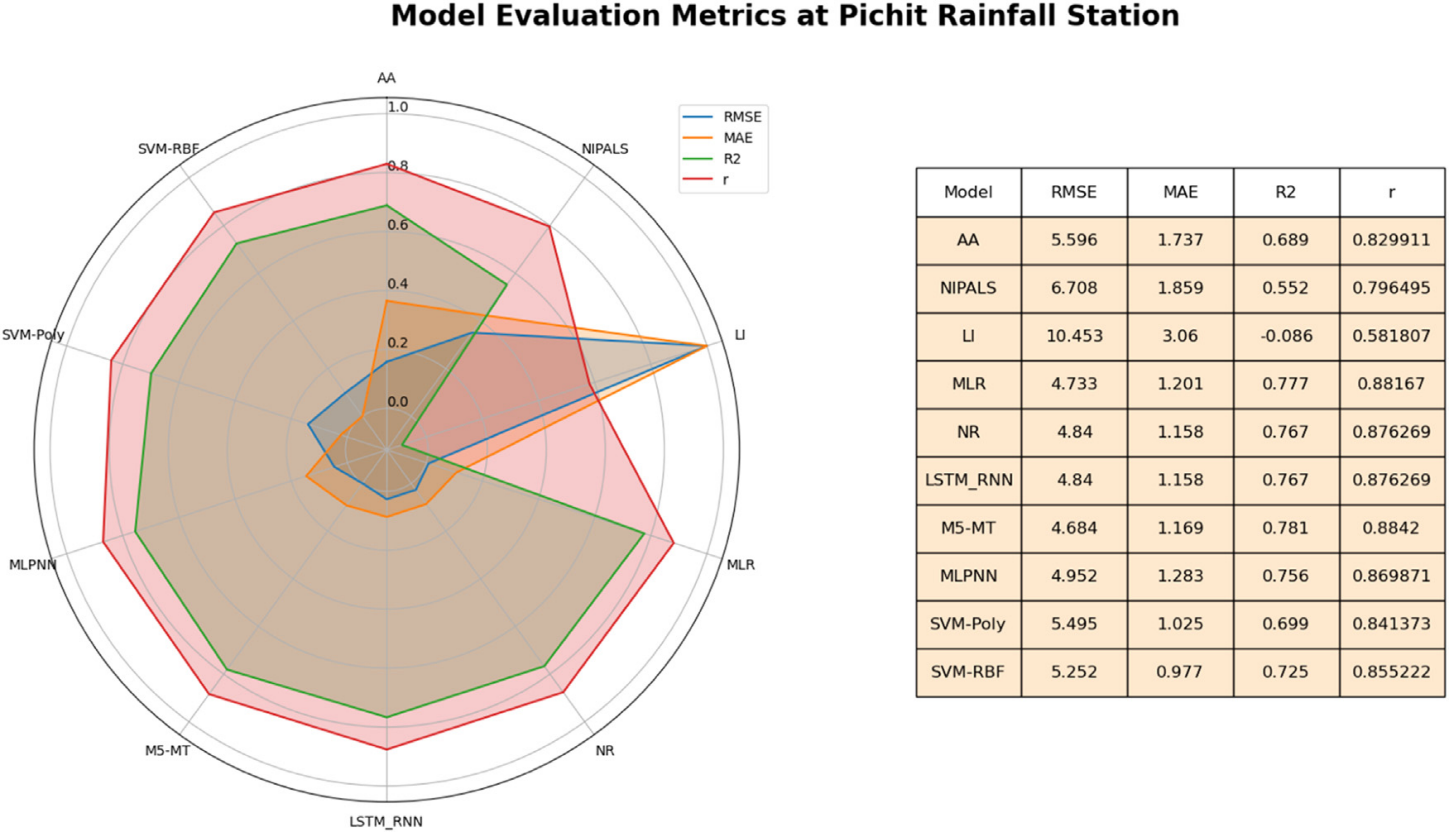
Performance of imputation methods at Pichit rainfall station.

At the Pichit station, the M5-MT method is the best approach for inputting missing daily rainfall data as compared to all STs and AITs. The M5-MT method showcases favorable performance, demonstrating a low MAE of 1.170, RMSE of 4.684, a high R^2^ value of 0.78, and a correlation (r) of 0.884 (Fig. 9). These results indicate that M5-MT provides accurate imputations and explains approximately 78.2 % of the variance in the imputed data. Among STs, MLR exhibited good results with R^2^ of 0.78, RMSE of 4.733, MAE of 1.201 and r of 0.882. Comparatively, other methods such as AA, NIPALS, LI, MLPNN, SVM-Poly, and SVM-RBF exhibit higher MAE and RMSE values, implying relatively larger imputation errors. Furthermore, these methods display lower R^2^ values, indicating a reduced ability to explain the variance in the imputed data.

The radar map highlights the relevance of M5-MT at the Pichit rainfall station since it stands out due to its data points stretching outward on many axes. According to this trend, M5-MT minimizes MAE and RMSE while obtaining a commendable R^2^. The robust performance of M5-MT suggests that it can make precise predictions and efficiently capture data variance at the Pichit station. The MLR method's shape, which features data points stretching outward across different axes, is comparable to M5-MT's shape on the radar map. This pattern shows that MLR can provide reliable fits (high R^2^), few mistakes (low MAE and RMSE), and accurate forecasts. Overall, M5-MT stands out as a viable method at the Pichit rainfall station.

**Fig. 10:**
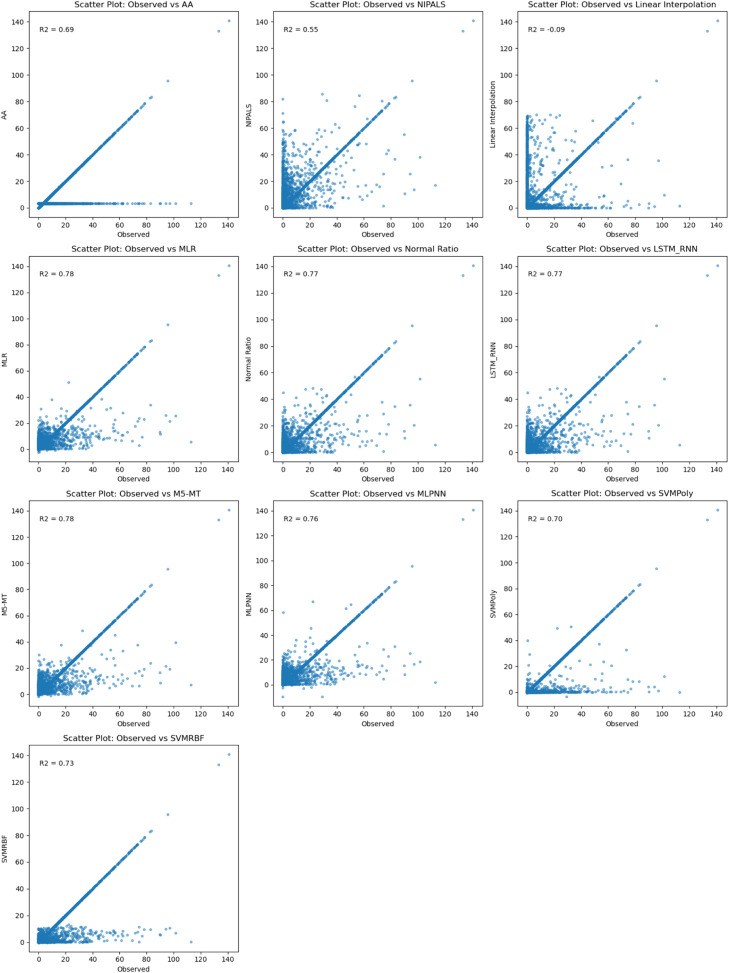
Comparison between observed and results of imputation method at Pichit rainfall station.

Fig. 10 highlights that the M5-MT outperforms them accurately and captures the underlying rainfall patterns. The MLR, NR, and LSTM-RNN methods also show favorable R^2^ values of 0.78, 0.77,and 0.77 respectively, indicating their effectiveness in imputing missing data. However, MLR and M5-MT achieves a slightly higher R^2^ value, suggesting a better fit of the imputation model to the actual rainfall patterns at Pichit rainfall station. Therefore, based on the results, the MLR and M5-MT methods are recommended as the best approach for estimating missing rainfall data at the Pichit rainfall station.

**Fig. 11:**
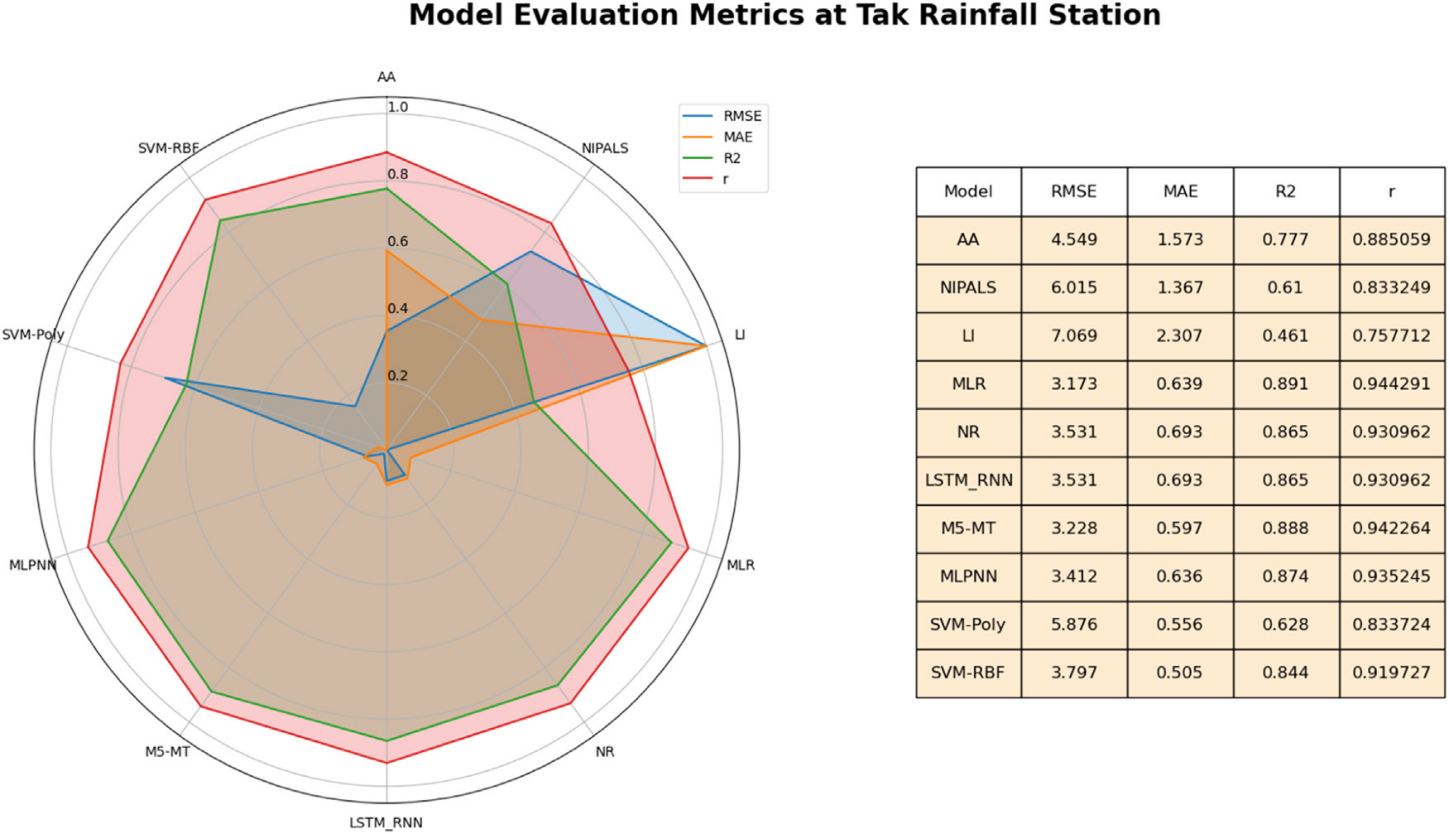
Performance of imputation methods at Tak rainfall station.

It can be seen in Fig. 11 at the Tak rainfall station that the MLR method is the best approach for inputting missing daily rainfall data at the Tak rainfall station. It exhibits exceptional performance, yielding the lowest MAE of 0.640, RMSE of 3.173, and R^2^ of 0.892. These results indicate that MLR provides highly accurate imputations and explains approximately 89.2 % of the variance in the imputed data. The M5-MT method also exhibits strong performance, with the lowest MAE of 0.597, RMSE of 3.228, and the highest R^2^ value of 0.888 among all the evaluated methods. Compared with other methods such as AA, NIPALS, LR, MLPNN, SVM-Poly, and SVM-RBF, they demonstrate higher MAE and RMSE values, implying larger imputation errors.

One method that shines prominently on the radar map is MLR. With data points extending outward on multiple axes, MLR showcases an exceptional performance profile. Similar patterns emerge for NR and LSTM-RNN, displaying outward extensions primarily on the MAE and RMSE axes. It indicates their proficiency in producing accurate predictions with relatively low errors. However, the slightly shorter extension on the R^2^ axis suggests that while they capture variability, their predictive strength might not be as robust as MLR. M5-MT follows a shape akin to MLR, with data points extending outward across multiple axes. This signifies M5-MT's commendable performance in generating accurate predictions, demonstrating a robust fit (high R^2^), and delivering lower errors (low MAE and RMSE). In summary, based on the results, the MLR and M5-MT methods are recommended as the most effective approach for imputing missing daily rainfall data in this study. While other methods excel in specific facets, these three consistently deliver accurate predictions, a robust fit to the data, and relatively low errors. The Radar Map's insights guide decision-making, aiding in selecting a method that aligns harmoniously with the study's research goals and contextual nuances.

**Fig. 12:**
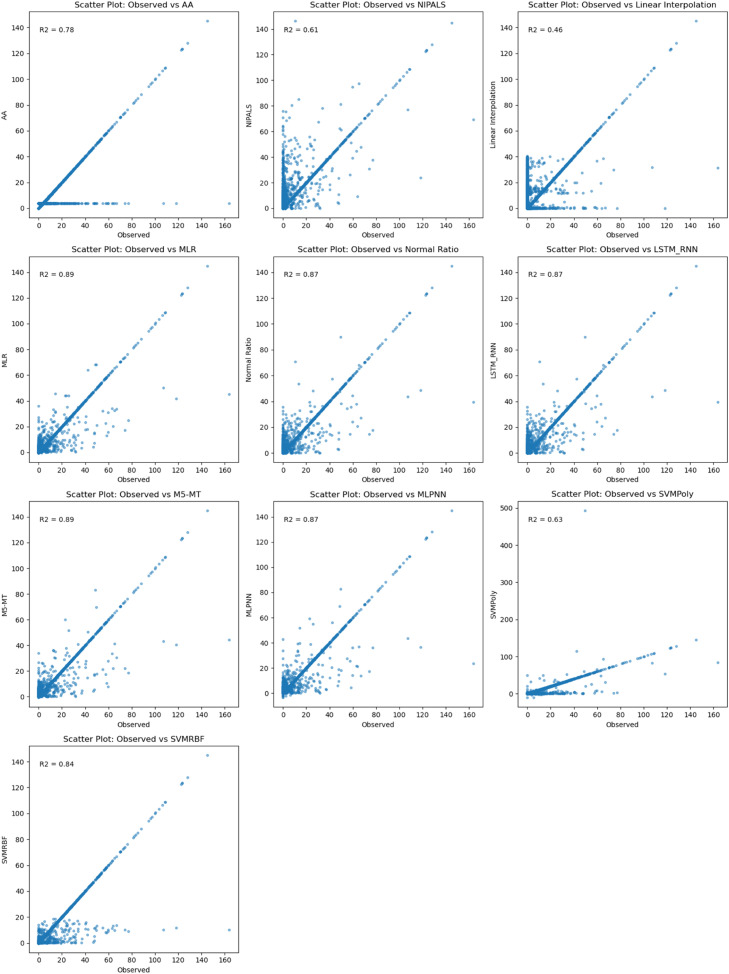
Comparison between observed and results of the imputation method at Tak rainfall station.

In Fig. 12, based on the R^2^ results between observed and imputed values by all methods, MLR and M5-MT can be considered the top-performing models for estimating missing rainfall data at Tak Rainfall station. These models provide reliable and accurate imputations, while Normal, LSTM-RNN, and MLPNN also demonstrate favorable performance. Researchers and practitioners can confidently utilize MLR, M5-MT, and other effective models to improve the accuracy of hydrological analyses and related studies at Tak Rainfall station.

**Fig. 13:**
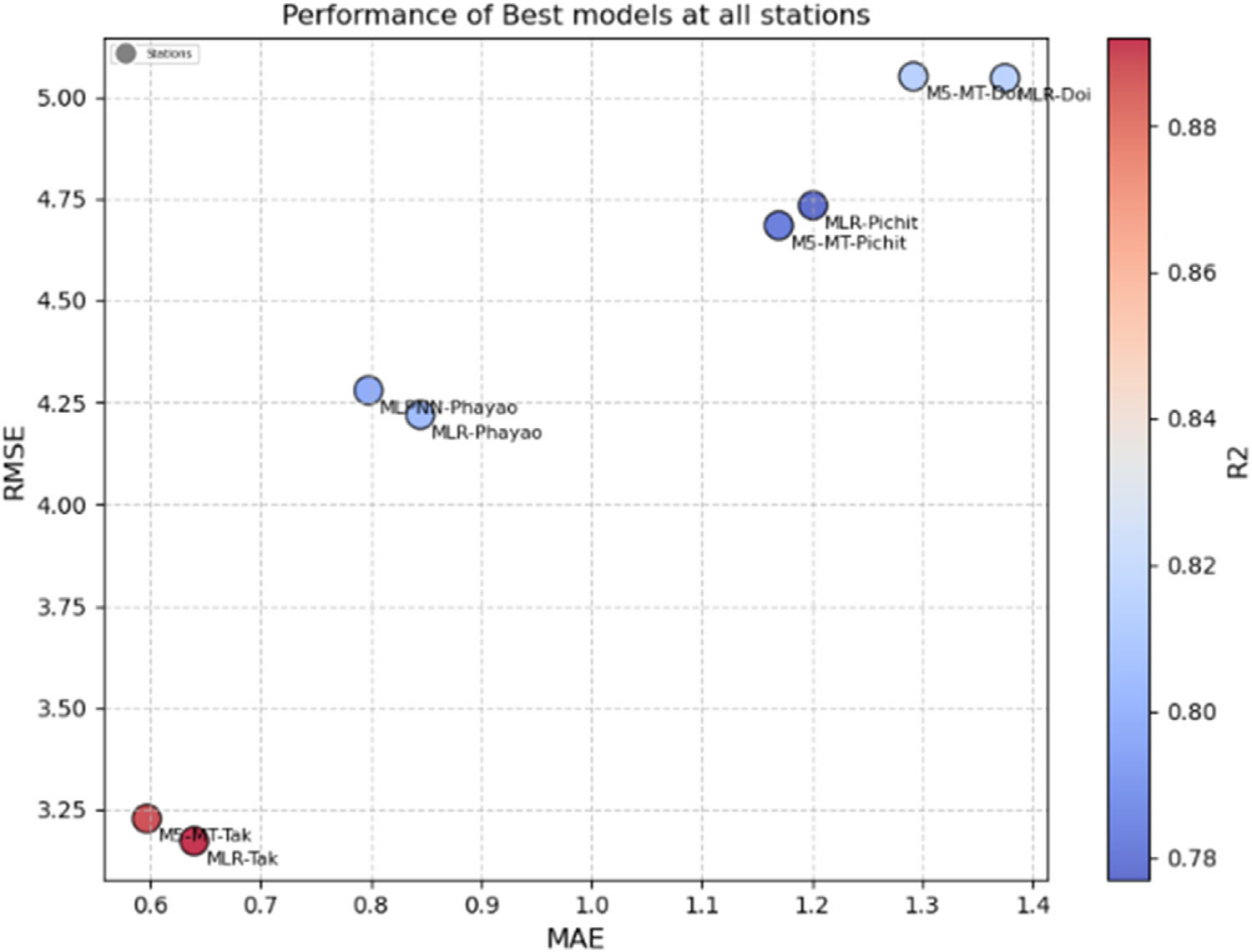
Scatter diagram between RMSE vs. MAE vs. R^2^ for best methods from all target stations.

In Fig. 13, the overall performance of best imputation models is presented which shows that MLR and M5-MT perform similarly at the Doi Ang Khang station, demonstrating moderate predictive accuracy. These models can explain approximately 81.5 % (MLR) and 81.4 % (M5-MT) of the variance in the data, respectively. The results suggest that both models are suitable for predicting at this station, but there is no significant difference in their performance. At the Phayao station, the MLPNN outperforms the MLR. The models' R^2^ values are also similar, with the MLR explaining around 79.8 % of the variance and the MLPNN explaining approximately 80.4 %. These results indicate that the MLPNN model offers slightly improved predictive accuracy over the MLR model at the Phayao station, making it a preferable choice for imputation in this specific location.

Similarly, at the Tak station, the MLR exhibits outstanding predictive accuracy. It achieves a significantly lower MAE of 0.640 and RMSE of 3.173 compared to the M5-MT, which yields an MAE of 0.597 and RMSE of 3.228. At the Pichit station, the MLR demonstrates moderate predictive accuracy for air quality assessment. It achieves an MAE of 1.201 and an RMSE of 4.733. The MLR model's R^2^ value of 0.777 suggests that it can explain approximately 77.7 % of the variance in the air quality data at this station. In contrast, the M5-MT model performs similarly to the MLR model at the Pichit station. It achieves a slightly improved MAE of 1.170 and RMSE of 4.684. The M5-MT model's R^2^ value of 0.782 indicates that it can explain around 78.2 % of the variance rainfall data. While both models offer acceptable predictive accuracy, neither model stands out as a clear winner. The results suggest that both the MLR and M5-MT models can provide reasonable predictions at the Pichit station, with the M5-MT model showing slight improvements in accuracy compared to the MLR model.

In the broader context of the study encompassing various STs and AITs, MLR emerged as a notably proficient method, as evidenced by its commendable performance with respect to key evaluation metrics such as R^2^, RMSE, MAE, and r. MLR consistently exhibited commendable performance across all designated rainfall stations, effectively harnessing the interrelatedness between the target station's data and that of its proximate counterparts, thereby yielding outcomes of heightened accuracy and precision.

## Discussion

The current investigation aims to propose an appropriate approach for imputing missing daily rainfall data at observation stations of the TMD, specifically in regions characterized by low inter-station correlation in Thailand. To achieve this objective, ten distinct imputation methods sourced from relevant literature were applied, and their performances were rigorously compared through statistical evaluation. This study draws inspiration from analogous research endeavors undertaken in diverse geographical regions across the globe. For example, Caldera et al. [52] evaluated ten different methods for filling gaps in data within a mountainous river basin in Sri Lanka. The study's findings indicate that both probabilistic and linear regression methods demonstrate strong performance when applied to target stations exhibiting a high correlation with a neighboring station. In contrast, the inverse distance squared and NR methods perform better for stations with lower correlation coefficients. It is worth noting that the MLR and weighted linear regression techniques necessitate the presence of nearby stations that exhibit a relatively strong correlation to achieve precise outcomes [52]. Yi Xun et al. compared an ANN to conventional methods for estimating missing rainfall data, including inverse distance weighting, linear regression, NR, and ordinary kriging. ANN outperformed conventional methods and was the superior method for determining missing data on rainfall in the Kelantan River Basin in Malaysia's tropical interior [1]. Shaharudin et al. focus on imputing missing rainfall data in hydrology and climatology modeling using a variety of imputation techniques: Replacing by Mean (RM), Nearest Neighbor, Random Forest (RF), NIPALS, and Markov Chain Monte Carlo. Utilizing monthly precipitation data from 24 rainfall stations in Yogyakarta, Indonesia, bootstrapping was used to estimate within-imputation standard errors. The performance evaluation based on RMSE revealed that the RF-Bootstrap (RF-B) method produced the most satisfactory results for estimating Yogyakarta, Indonesia's missing precipitation data [5]. Pinthong et al. compare ML and spatial interpolation techniques for estimating missing monthly rainfall data.

ML methods outperformed SI methods because they effectively address spatial limitations. Genetic programming yielded the highest ML performance, followed by SVR-rbf, SVR-poly, and RF. NR exhibited the best performance among SI methods, followed by correlation coefficient weighted, AA, and Inverse distance weighting. A correlation greater than 0.80 between the target and neighboring stations was necessary for applying SI methods [12]. Djerbouai utilized the LSTM deep neural network model to estimate missing monthly precipitation data in the K'sob basin, Algeria.

Through a trial-and-error process, the optimal architecture of the LSTM model was adjusted. The LSTM model outperformed traditional methods like inverse distance weighting and coefficient of correlation weighting methods in accuracy for estimating missing data [53]. Papailiou et al. ntroduce an ensemble approach using MLPNN to estimate daily missing rainfall data in the extended region of Chania, Greece. The methodology aims to create precipitation time series by utilizing data from nearby stations. The ANN ensembles demonstrated higher accuracy than the MLR model for handling missing data, although they required a longer processing time [54]. The current study utilized ten techniques for imputing the missing rainfall data from four specific target stations within each of the two climatic regions in Thailand. The study exclusively focused on the classification of climatic or ecological divisions, a previously examined solely by [1]. Before estimating daily rainfall missing data at the target stations, a thorough examination of the missingness mechanism for the missing rainfall data was undertaken, adhering to the guidelines outlined in reference [13].

It is worth noting that, based on the author's best knowledge, none of the prior studies have tested the mechanism of missingness for observation of the daily rainfall dataset in Thailand. This study thoroughly examined the existing literature to identify and select ten appropriate methods for the research objectives. The selection process considered the criteria of simplicity and performance in selected regions. The comprehensive analysis of various methodologies has facilitated increased adaptability in determining the optimal approach for estimating missing data in daily rainfall observations. A distinctive aspect of this study is utilizing radar maps or spider plots and scatter plots to represent the effectiveness of all methods visually. The methodology above yielded significant observations regarding the real situations at various stations in northern Thailand with incomplete data about 25–35 % of missingness, thereby impacting the effectiveness of certain techniques for estimating missing values. Although this study did not introduce innovative methodologies, it successfully incorporated various methodologies and comparison criteria alongside descriptive measures to estimate the absence of daily rainfall data. This undertaking establishes the groundwork for forthcoming scientific inquiries about the continuous collection of rainfall data, thereby fostering progress in the respective field.

## Conclusion and future directions

The present study investigated a range of statistical techniques (STs)s and artificial intelligence techniques (AITs) to impute missing daily rainfall data in northern Thailand. By assessing various performance metrics, it was determined that MLR exhibited favorable performance in accurately estimating missing daily rainfall data. AITs such as M5-MT performed well at some target stations, and it has ability to impute missing values at different regions. Also, LSTM-RNN and MLPNN also demonstrated promising outcomes. At the Doi Ang Khang station, it was observed that both the MLR and M5-MT models demonstrated comparable levels of predictive accuracy, with MLR explaining approximately 81.5 % of the variance and the M5-MT model explaining approximately 81.4 % of the variance. At the Phayao station, the MLPNN performs superior to MLR, exhibiting a marginal enhancement in predictive accuracy. The R^2^ values for MLPNN and MLR are approximately 80.4 % and 79.8 %, respectively. In a similar vein, the performance of MLR in terms of predictive accuracy at Tak station surpasses that of M5-MT, as evidenced by its notably lower MAE and RMSE values (0.640 and 3.173 for MLR, compared to 0.597 and 3.228 for M5-MT). At Pichit station, the MLR and M5-MT models demonstrate comparable levels of predictive accuracy, with R^2^ values of approximately 77.7 % and 78.2 %, respectively. Overall, the MLR technique stood out at all target stations as a recommended approach due to its ability to deliver good estimation results while offering a transparent mechanism and not necessitating prior knowledge for model creation.

• The findings above underscore the significance of employing suitable methodologies that align with the specific attributes of the station to guarantee efficient prediction and monitoring. Based on the findings of this study,subsequent research endeavors may explore the potential of hybrid methodologies that integrate the advantages of diverse approaches, including STs and AITs models.
• The utilization of hybrid methodologies has the potential to enhance the precision and resilience of imputing missing rainfall data. Integrating spatial analysis and including geographical factors in imputation models can improve the precision of rainfall data estimation.

## Funding

This research received no external funding.

## Data availability statement

Data used to support the study's findings can be obtained from the corresponding author upon request.

## CRediT authorship contribution statement

**Angkool Wangwongchai:** Conceptualization, Formal analysis, Investigation, Methodology, Software, Visualization, Writing – original draft. **Muhammad Waqas:** Conceptualization, Formal analysis, Investigation, Methodology, Validation, Writing – review & editing. **Porntip Dechpichai:** Conceptualization, Formal analysis, Supervision, Validation, Writing – review & editing, Funding acquisition. **Phyo Thandar Hlaing:** Conceptualization, Formal analysis, Supervision, Validation, Writing – review & editing. **Shakeel Ahmad:** Validation, Writing – review & editing, Formal analysis. **Usa Wannasingha Humphries:** Supervision, Validation, Project administration, Visualization.

## Declaration of Competing Interest

The authors declare that they have no known competing financial interests or personal relationships that could have appeared to influence the work reported in this paper.

## Data Availability

Data will be made available on request.
